# Uncovering the Critical Role of Cuproptosis in Wilson Disease: Insights Into Potential Therapeutic Targets

**DOI:** 10.1111/jcmm.70946

**Published:** 2025-11-13

**Authors:** Shan Tang, Feng Ren, Wei Hou, Zihao Fan, Yinkang Mo, Xianru Zhu, Yaling Cao, Ling Xu, Sujun Zheng

**Affiliations:** ^1^ First Department of Hepatology, Beijing Youan Hospital Capital Medical University Beijing China; ^2^ Beijing Institute of Hepatology/Beijing Youan Hospital Capital Medical University Beijing China

**Keywords:** cuproptosis, diagnostic biomarkers, differentially expressed genes, hub genes, therapeutic targets, Wilson disease

## Abstract

Wilson disease (WD) is an inherited disorder caused by ATP7B mutations, resulting in toxic copper accumulation primarily in the liver and brain. While copper‐induced hepatotoxicity is a hallmark of WD, the mechanisms linking copper overload to liver injury remain unclear. This study aimed to investigate the role of cuproptosis, a copper‐dependent form of regulated cell death, in WD pathogenesis and identify key cuproptosis‐related genes (CRGs). We utilised ATP7B−/− mice and HepG2 cells to model WD. Liver injury was assessed histologically and biochemically. Transcriptomic analysis identified differentially expressed CRGs, followed by machine learning (LASSO, SVM‐RFE) to identify key genes. Functional enrichment and protein validation were performed. Candidate biomarkers were evaluated in WD patient serum and confirmed in the mouse model. ATP7B−/− mice showed marked hepatocellular injury with elevated AST, ALT and LDH. Cuproptosis markers (FDX1, DLST, DLAT, LIAS) were upregulated in both liver tissue and HepG2 cells. Copper exposure decreased cell viability and increased LDH release, exacerbated by Elesclomol and alleviated by Tetrathiomolybdate. Transcriptomics revealed Lox, App, Afp, Alb, Gpc1, Gls were central hub genes. Importantly, SiRNA knockdown of Gpc1, Gls, Lox and App alleviated cuproptosis, supporting their key roles in cuproptosis. Cuproptosis plays a critical role in copper‐induced liver injury in WD. Key mediators identified include Gpc1, Gls, Lox and App, which were validated as potential therapeutic targets. These findings provide new insights into the molecular mechanisms underlying WD and may inform the development of targeted treatment strategies.

## 1 Introduction

Wilson disease (WD) is a rare autosomal recessive disorder of copper metabolism, resulting from mutations in the ATP7B gene. It has an estimated incidence of 1 in 30,000 individuals, with a higher carrier frequency of approximately 1 in 90 [1]. WD is asymptomatic at birth. However, as copper gradually accumulates in various organs and tissues—especially the liver and brain—affected individuals start to manifest a wide range of symptoms. These often include hepatic, neurological and psychiatric disorders [2]. If left untreated, the disease can be fatal, with a mortality rate of approximately 5.0%–6.1% [3]. Early intervention with chelating agents or zinc salts can effectively manage copper levels, greatly improving prognosis and preserving both liver function and overall well‐being [4].

Current pharmacological therapies for WD include chelators (d‐penicillamine and trientine) that reduce copper levels by increasing urinary excretion, and zinc salts that decrease copper uptake from the gastrointestinal tract [5]. While these treatments can control symptoms, they do not cure the disease and have limitations. d‐penicillamine, for example, is associated with severe side effects like bone marrow toxicity, hypersensitivity reactions, nephrotoxicity, lupus‐like syndrome and dermatological issues, affecting about 30% of patients, leading to discontinuation [6]. Additionally, psychiatric symptoms are often poorly managed and in some cases, may worsen during treatment [7]. What's more, a study revealed that only 74.1% of patients adhere to treatment over a mean follow‐up of 11.7 years, which is linked to worse clinical outcomes [8]. In this context, exploring the molecular mechanisms of WD from a novel perspective is crucial for developing more effective treatment strategies. However, a significant gap remains in our understanding of the precise molecular mechanisms underlying copper‐induced toxicity in WD.

Recent research has introduced a novel form of cell death called cuproptosis, which is specifically induced by excess copper accumulation [9]. This process can be summarised as follows: excessive free copper in cells binds directly to lipoylated proteins in the tricarboxylic acid cycle, forming toxic polymers. This interaction disrupts iron–sulfur cluster proteins, ultimately resulting in cell death [10]. Unlike traditional forms of cell death, such as apoptosis, cuproptosis is characterised by mitochondrial disruption and the accumulation of copper within cells, leading to mitochondrial dysfunction [11]. This process represents a new and emerging area of research in the context of copper‐related diseases.

In WD, the liver serves as the primary site of copper accumulation due to defective copper transport. While extensive research has examined the impact of oxidative stress and mitochondrial dysfunction caused by copper overload [12, 13, 14], the specific role of cuproptosis in WD remains largely unexplored. Given that mitochondrial injury is a hallmark of WD pathophysiology, it is likely that cuproptosis plays a crucial role in liver damage. Therefore, investigating the role of cuproptosis could provide valuable insights into the molecular mechanisms driving WD and potentially uncover novel therapeutic targets.

In this study, we aimed to investigate the role of cuproptosis in liver injury in WD. Using both in vivo and in vitro models, we first examined whether copper overload activates the cuproptosis pathway, contributing to liver damage. Next, we explored its role in cellular injury by selectively activating and inhibiting this pathway. Finally, through transcriptome sequencing, we identified key differentially expressed cuproptosis‐related genes and analysed their involvement in various biological processes. To validate these findings, we assessed the expression of these key genes in the ATP7B−/− mouse model, confirming their relevance to the disease. We further performed siRNA knockdown experiments in ATP7B−/− HepG2 cells to validate their functional roles. This study is significant in revealing a previously unrecognised mechanism, identifying cuproptosis as a key contributor to liver dysfunction in WD. Furthermore, by identifying key genes associated with this process, we aim to provide novel molecular targets for therapeutic intervention, enhance the management of WD and deepen our understanding of copper toxicity at the cellular level.

## 2 Materials and Methods

### 2.1 WD Mouse Model and Sample Collection

In this study, ATP7B−/− (WD) mice on a C57BL/6 background were generated using CRISPR/Cas9 technology and were kindly provided by Professor Jian Huang. Age‐matched C57BL/6 wild‐type (WT) mice served as controls. All mice were housed in a specific pathogen‐free (SPF) environment and were provided with food and autoclaved water from WQJX Biotechnology, with no additional treatments administered.

For further analysis, four 20‐week‐old WD mice (approximately 25 g each) and four WT mice were selected. All experimental procedures were conducted under the supervision of the Experimental Animal Ethics Committee of Beijing You'an Hospital, Capital Medical University.

### 2.2 RNA Sequencing

Total RNA was extracted from ATP7B−/− and C57BL/6 mice, followed by quality control to assess RNA quantity, purity and integrity. A cDNA library with an average insert size of 300 ± 50 bp was prepared from approximately 1 μg of total RNA. Sequencing was performed on an Illumina NovaSeq 6000 platform using a 2 × 150 bp paired‐end configuration. Differentially expressed genes (DEGs) were identified with a fold change > 2 and a *p*‐value < 0.05. Cuproptosis‐associated genes (CRGs) were selected based on prior literature [15].

### 2.3 Screening of Cuproptosis‐Associated Genes in WD

Differentially expressed cuproptosis‐related genes (DE‐CRGs) were first identified using Venn diagrams, followed by the construction of heatmaps to visualise their expression patterns. The relationships between the DE‐CRGs were analysed using Pearson correlation coefficients, with the results visualised through the ‘corrplot’ function in R (version 4.1.2). To explore the functional roles of these genes, Gene Ontology (GO) and Kyoto Encyclopedia of Genes and Genomes (KEGG) pathway enrichment analyses were performed using the ClusterProfiler package in R. Additionally, the genes were uploaded to the STRING database (https://cn.string‐db.org/) to generate a protein–protein interaction (PPI) network, which was visualised using Cytoscape 3.9.1.

### 2.4 Constructing Diagnostic Models

To identify the most significant DE‐CRGs, we utilised the least absolute shrinkage and selection operator (LASSO) model and support vector machine recursive feature elimination (SVM‐RFE). LASSO, which applies an *ℓ*1 penalty to produce a sparse solution, was implemented using the ‘glmnet’ package in R. The optimal lambda value was chosen as the key parameter to optimise model performance.

### 2.5 Cell Culture

The ATP7B knockout model of the human HepG2 cell line was established by CRISPR‐U‐mediated genome engineering (Supporting Information). Guide RNA used in this work was shown in Table S1. In addition, we performed dot plot and GC content distribution analyses to verify the suitability of the selected target region and gRNA design. Overview of GC content distribution was shown in Figure S1. HepG2 cells were cultured in Dulbecco's Modified Eagle Medium (DMEM) (Biochrom AG, Berlin, Germany) supplemented with 10% fetal bovine serum (Biochrom AG) and 2 mM l‐Glutamine (PAA Laboratories GmbH, Pasching, Austria) at 37°C under 5% CO2 in a humidified incubator.

### 2.6 Cell Viability Assay

ATP7B−/− HepG2 cells were seeded into 96‐well plates and treated as required. After treatment, the culture medium was carefully removed, and 10 μL of Cell Counting Kit‐8 (CCK‐8; Dojindo, Kumamoto, Japan) reagent was added to each well. The plates were then incubated at 37°C for 20 min. Absorbance at 450 nm was measured using a Bio‐Rad microplate spectrophotometer, and cell viability was determined based on the absorbance readings.

### 2.7 Lactate Dehydrogenase (LDH) Content Detection

LDH levels were assessed using the Lactate Dehydrogenase (LDH) Cytotoxicity Colorimetric Assay Kit (Elabscience, Wuhan, China) following the manufacturer's instructions.

### 2.8 Haematoxylin and Eosin (H&E) Staining

Liver tissue sections, fixed in formalin and embedded in paraffin, were stained with Masson's Trichrome (MT) (Gefan, China) following the manufacturer's instructions. To assess hepatic collagen distribution, five randomly selected fibrotic septa from the left and right liver lobes of four mice per group were analysed. Collagen content was quantified by calculating the percentage of the stained area in each section.

### 2.9 Determination of Biochemical Parameters in Serum

Liver injury markers, including alanine aminotransferase (ALT), aspartate aminotransferase (AST) and LDH, were measured in serum using Fujifilm Dri‐Chem Slides and the Fuji Dri‐Chem 4000i Automated Clinical Chemistry Analyser, following the manufacturer's instructions. Results are expressed as figures per IU/L, as described.

### 2.10 Quantitative Real‐Time PCR

The mRNA expression levels of target genes were analysed by real‐time quantitative PCR. RNA was extracted from cultured cells and liver tissues using TRIzol reagent (Invitrogen, Carlsbad, CA, USA). The RNA was then reverse‐transcribed into cDNA using the AMV reverse transcriptase system (TaKaRa, Dalian, Liaoning, China). cDNA amplification was performed with the SYBR Green reaction mixture (TaKaRa, Dalian, Liaoning, China) on an ABI StepOne Plus system (Applied Biosystems). Primers (listed in Table S2) were designed using Primer 5.0 software. Relative gene expression was normalised to the housekeeping gene *GAPDH*.

### 2.11 Western Blot

RIPA lysis buffer (Solarbio) supplemented with protease inhibitor (Solarbio) and phosphatase inhibitor (Beijing Aoqing Biotechnology Co. Ltd., Beijing, China) was used to extract the total protein from cultured cells and liver tissues. The protein concentration was determined using the BCA protein quantitative kit (Solarbio) according to the manufacturer's instruction. Proteins were subjected to SDS‐PAGE and transferred to a polyvinylidene fluoride (PVDF) membrane (Millipore). The membranes were blocked with 5% BSA in a horizontal shaker at room temperature for 60 min, and then incubated overnight with the following primary antibodies at 4°C: LIAS (YT8133) rabbit pAb (1:2000; Immunoway), DLAT (PA5‐120279) rabbit pAb (1:2000; Thermo Scientific), DLST (ab187699) rabbit pAb (1:2000; Abcam) and β‐actin (1:5000; Immunoway). After TBST washing, the membranes were incubated with the corresponding secondary antibodies, HRP‐conjugated Goat Anti‐Rabbit IgG (H+L) (1:10,000; Jackson, America), at room temperature for 60 min. Luminol ECL reagent (Millipore) was used to visualise protein bands.

### 2.12 SiRNA Transfection

ATP7B−/− HepG2 cells were seeded in 48‐well plates and transfected with siRNAs targeting GPC1, GLS, LOX and APP using Lipofectamine 3000 (Thermo Fisher) according to the manufacturer's instructions. After 24 h of transfection, cells were treated with 600 μM copper to induce cuproptosis. Subsequently, mRNA levels of cuproptosis‐related genes (DLAT, DLST, LIAS) were quantified by real‐time PCR. Sequences of all siRNAs are listed in Table S3.

### 2.13 Statistical Analysis

Statistical analyses were conducted using GraphPad Prism 8.0 software. Data are expressed as mean ± standard error of the mean (SEM) and were tested for normality and homogeneity of variance. Differences between two groups were assessed using a Student's *t*‐test. *p*‐value of ≤ 0.05 was considered statistically significant. For comparisons involving multiple groups, *p* values were adjusted using the Benjamini‐Hochberg false discovery rate (FDR) correction to control for type I error. Adjusted *p* values (also reported as *q* values) ≤ 0.05 were considered statistically significant.

## 3 Results

### 3.1 Copper Overload Induced Liver Injury and Cuproptosis in ATP7B−/− Mice

**FIGURE 1:**
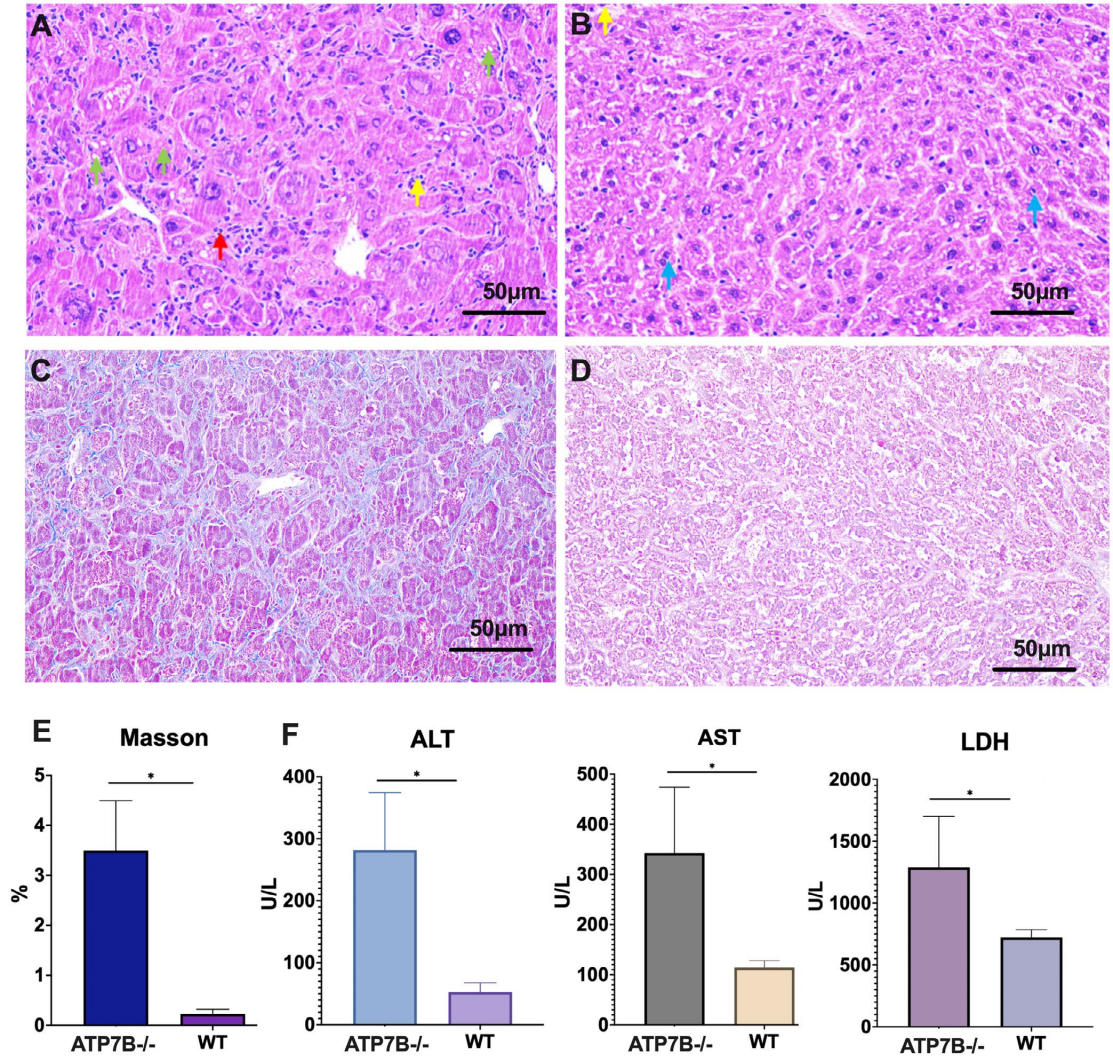
Liver tissue of ATP7B−/− mice exhibited inflammation and necrosis. (A) H&E staining of liver tissue from 20‐week‐old ATP7B−/− mice reveals significant liver damage, including hepatocyte swelling, mild fatty degeneration and cytoplasmic vacuolation (green arrows). Hepatic cords appear compact, with no evident sinusoidal dilation. Inflammatory cell infiltration (red arrows) and hepatocyte necrosis, characterised by nuclear fragmentation, condensation and hyperchromasia (yellow arrows), are observed. (B) H&E staining of liver tissue from 20‐week‐old WT mice shows well‐organised hepatocytes with minimal edema and lightly stained cytoplasm (blue arrow). Hepatic cords are arranged tightly, with slight sinusoidal dilation (yellow arrow) and no significant inflammatory infiltration. (C) Masson staining of liver tissue in ATP7B−/− mice. (D) Masson staining of liver tissue in WT mice. (E) Masson staining demonstrates significantly increased liver fibrosis in ATP7B−/− mice compared to WT mice. (F) Serum biochemical analysis indicates significantly elevated ALT, AST and LDH levels in ATP7B−/− mice, reflecting severe liver inflammation and damage. ALT, alanine aminotransferase; AST, aspartate aminotransferase; ATP7B−/−, ATP7B knockout mice; LDH, lactate dehydrogenase; WT, wild‐type control mice; *p < 0.05.

First, we aimed to confirm that copper overload induces liver injury and cuproptosis in ATP7B−/− mice. H&E staining revealed significant liver damage in ATP7B−/− mice, with many hepatocytes showing swelling, a small number undergoing fatty degeneration and small round vacuoles visible in the cytoplasm (indicated by green arrows). The hepatic cords were tightly arranged, and the hepatic sinusoids did not show obvious expansion. Inflammatory cell infiltration was evident (indicated by red arrows), and some hepatocytes exhibited necrosis, with fragmented, condensed and darkly stained nuclei (indicated by yellow arrows) (Figure 1A). No notable inflammatory infiltration was observed in the healthy control mice (Figure 1B). Masson staining further demonstrated that liver fibrosis was significantly more pronounced in ATP7B−/− mice compared to controls (Figure 1C,D). Additionally, serum levels of AST, ALT and LDH were significantly elevated in ATP7B−/− mice, with statistically significant differences compared to control mice, confirming that WD mice experienced substantial liver injury.

**FIGURE 2:**
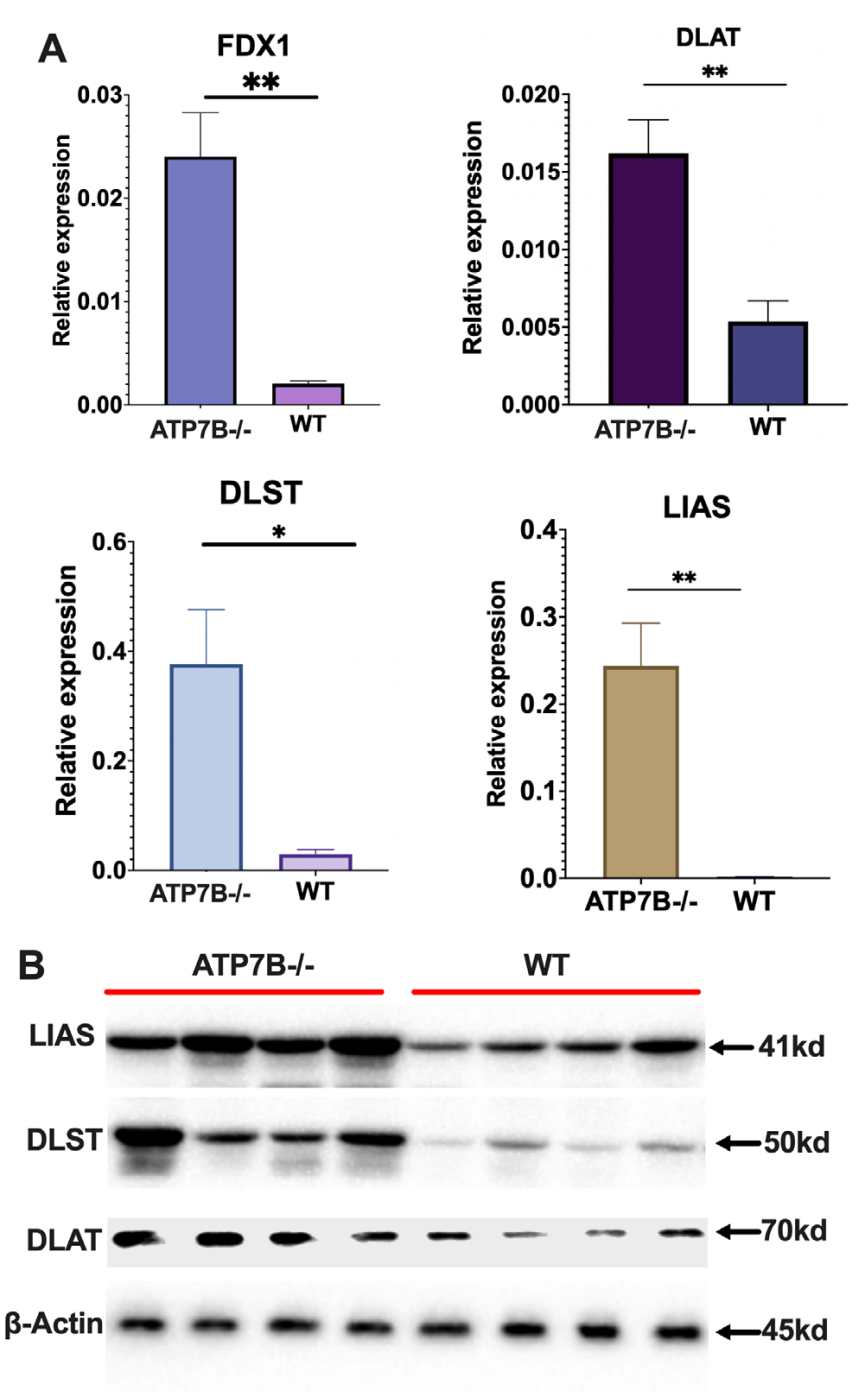
Cuproptosis occurs in ATP7B−/− mice. (A) mRNA expression levels of FDX1, DLAT, DLST and LIAS in ATP7B−/− and wild‐type (WT) mice. (B) Protein expression levels of LIAS, DLST and DLAT in ATP7B−/− and WT mice. ATP7B−/−, ATP7B knockout mice; WT, wild‐type control mice; *p < 0.05, **p < 0.01.

To further confirm the occurrence of cuproptosis in ATP7B−/− mice, we analysed the expression of key cuproptosis‐related markers. Compared to control mice, the mRNA levels of FDX1, DLST, DLAT and LIAS were significantly upregulated (Figure 2A). Western blot analysis further demonstrated a notable increase in DLST, DLAT and LIAS protein expression in ATP7B−/− mice (Figure 2B). These findings suggest that copper overload in WD induces liver damage, potentially through the activation of cuproptosis.

### 3.2 Copper Overload Induces Cuproptosis in ATP7B−/− HepG2 Cells

**FIGURE 3:**
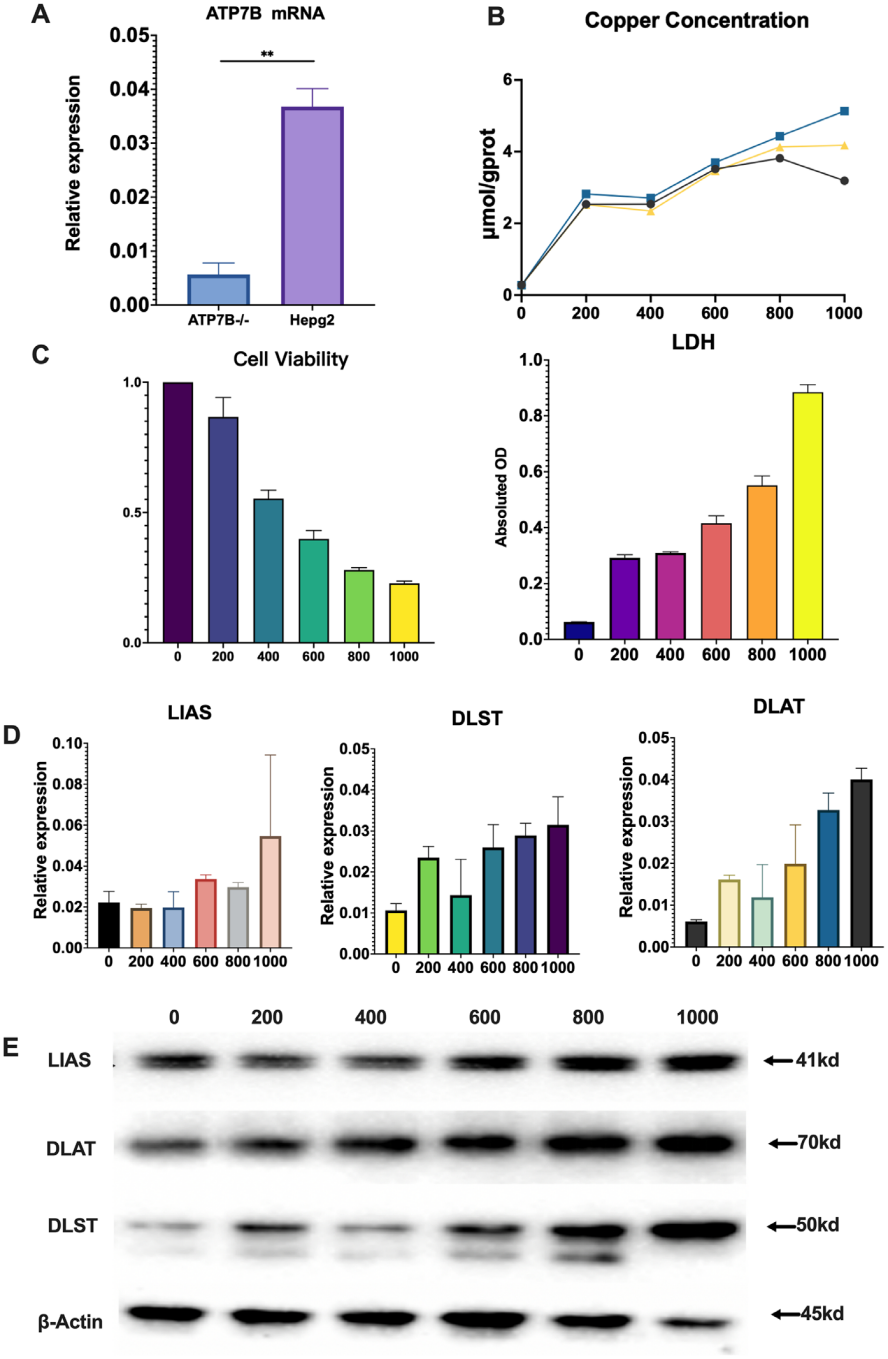
Copper overload induces cuproptosis in ATP7B−/− HepG2 cells. (A) mRNA expression levels of ATP7B in ATP7B−/− HepG2 cells and wild‐type HepG2 cells. (B) Intracellular copper concentration in ATP7B−/− HepG2 cells following treatment with varying concentrations of CuSO_4_. (C) Cell viability and LDH levels in ATP7B−/− HepG2 cells after exposure to different concentrations of CuSO_4_. (D) The mRNA expression levels of cuproptosis‐related genes (LIAS, DLST and DLAT) in ATP7B−/− HepG2 cells following CuSO_4_ treatment. (E) The protein expression levels of LIAS, DLST and DLAT in ATP7B−/− HepG2 cells after treatment with different concentrations of CuSO_4_. ATP7B−/−, ATP7B−/− HepG2 cells; HepG2, wild‐type HepG2 cells.

First, PCR results demonstrated that the ATP7B mRNA expression level in ATP7B−/− HepG2 cells was significantly reduced compared to wild‐type HepG2 cells, confirming the successful establishment of the ATP7B−/− HepG2 cell model (Figure 3A). To investigate whether copper overload induces cuproptosis in vitro, we measured intracellular copper concentration in cells treated with CuSO_4_ at concentrations ranging from 0 to 1000 μM. The results showed a positive correlation between intracellular copper levels and CuSO_4_ concentration (Figure 3B).

Next, we assessed cell viability using the CCK‐8 assay and measured LDH levels as indicators of cell damage. The data revealed that increasing concentrations of CuSO_4_ led to a significant decrease in cell viability of ATP7B−/− HepG2 cells, accompanied by a gradual increase in LDH levels (Figure 3C), suggesting that cell damage was positively correlated with CuSO_4_ concentration.

Additionally, quantitative PCR analysis showed that the mRNA levels of cuproptosis‐related genes, including LIAS, DLST and DLAT, exhibited an upward trend in CuSO_4_‐treated ATP7B−/− HepG2 cells (Figure 3D). Similarly, western blot analysis revealed that the protein expression of cuproptosis markers (LIAS, DLST and DLAT) was significantly increased following CuSO_4_ treatment (Figure 3E). These results confirm that copper overload induces cuproptosis in ATP7B−/− HepG2 cells.

### 3.3 Cuproptosis Promotes Cell Damage in ATP7B −/− HepG2 Cells

**FIGURE 4:**
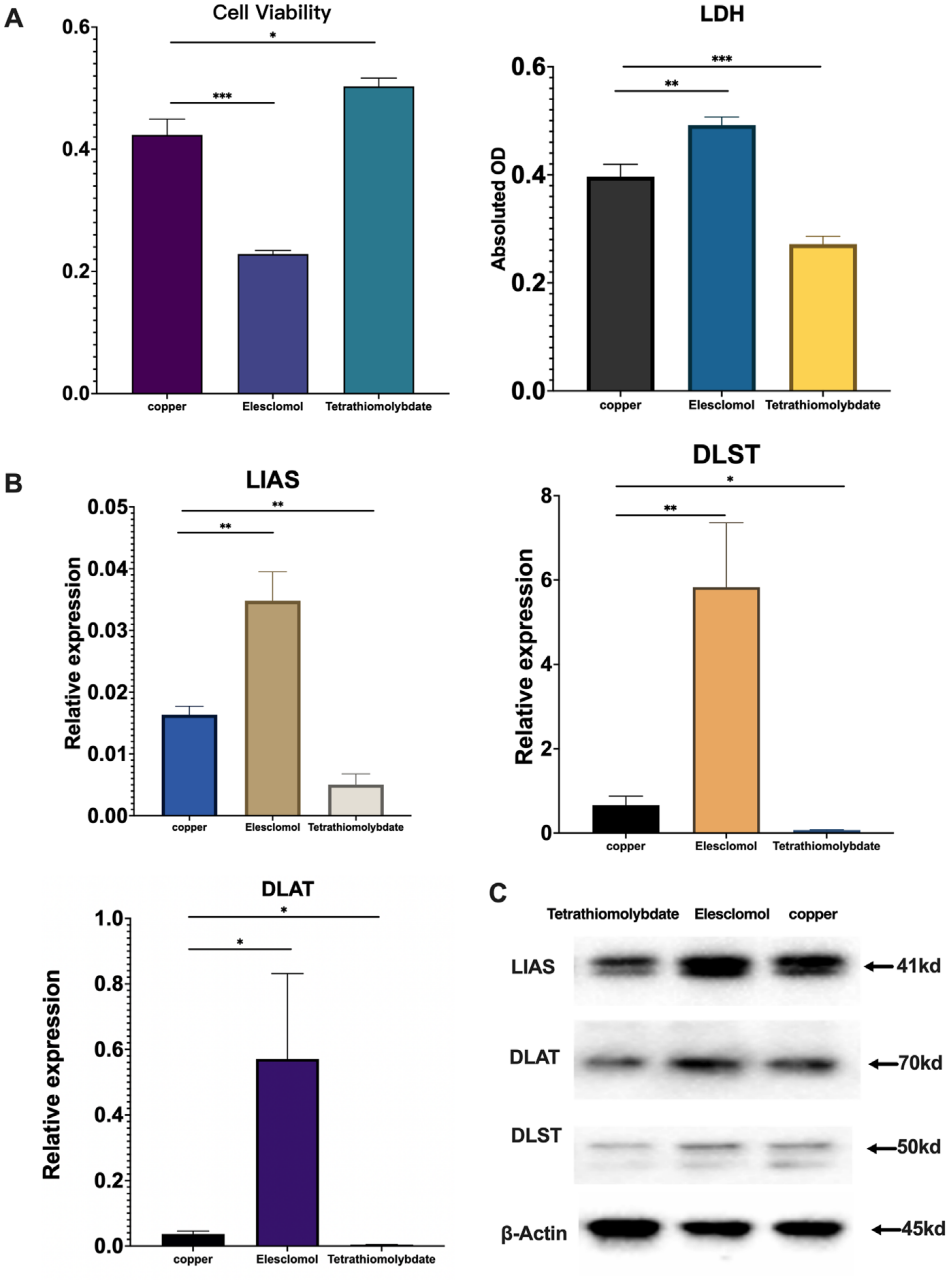
Inhibiting cuproptosis alleviates copper‐induced cell damage in ATP7B−/− HepG2 cells. (A) Cell viability and LDH levels in ATP7B−/− HepG2 cells following treatment with the cuproptosis activator Elesclomol and the inhibitor Tetrathiomolybdate. (B) mRNA expression levels of LIAS, DLST and DLAT in ATP7B−/− HepG2 cells after treatment with Elesclomol and Tetrathiomolybdate. (C) Protein expression levels of LIAS, DLST and DLAT in ATP7B−/− HepG2 cells following treatment with Elesclomol and Tetrathiomolybdate. Copper: ATP7B−/− HepG2 cells treated with 600 μmol/L CuSO_4_ alone; Elesclomol: ATP7B−/− HepG2 cells co‐treated with 600 μmol/L CuSO_4_ and the cuproptosis activator Elesclomol; Tetrathiomolybdate: ATP7B−/− HepG2 cells co‐treated with 600 μmol/L CuSO_4_ and the cuproptosis inhibitor tetrathiomolybdate. *p < 0.05; **p < 0.01; **p < 0.001.

To further explore whether copper‐induced cell damage is mediated by cuproptosis, we treated ATP7B−/− HepG2 cells with CuSO_4_ in the presence of the cuproptosis activator Elesclomol and the inhibitor Tetrathiomolybdate. The results showed that Elesclomol treatment significantly reduced cell viability and exacerbated cell damage in CuSO_4_‐treated ATP7B−/− HepG2 cells. In contrast, Tetrathiomolybdate treatment markedly improved cell viability and alleviated cell damage (Figure 4A). Furthermore, the mRNA and protein expression levels of cuproptosis‐related markers (LIAS, DLST and DLAT) were significantly upregulated in cells treated with CuSO_4_ and Elesclomol, whereas they were notably downregulated in cells treated with CuSO_4_ and Tetrathiomolybdate (Figure 4B,C). These findings indicate that cuproptosis plays a crucial role in copper‐induced injury in ATP7B−/− HepG2 cells and its inhibition can alleviate cell damage.

### 3.4 Screening of Potential Cuproptosis‐Associated Genes in ATP7B−/− Mice

**FIGURE 5:**
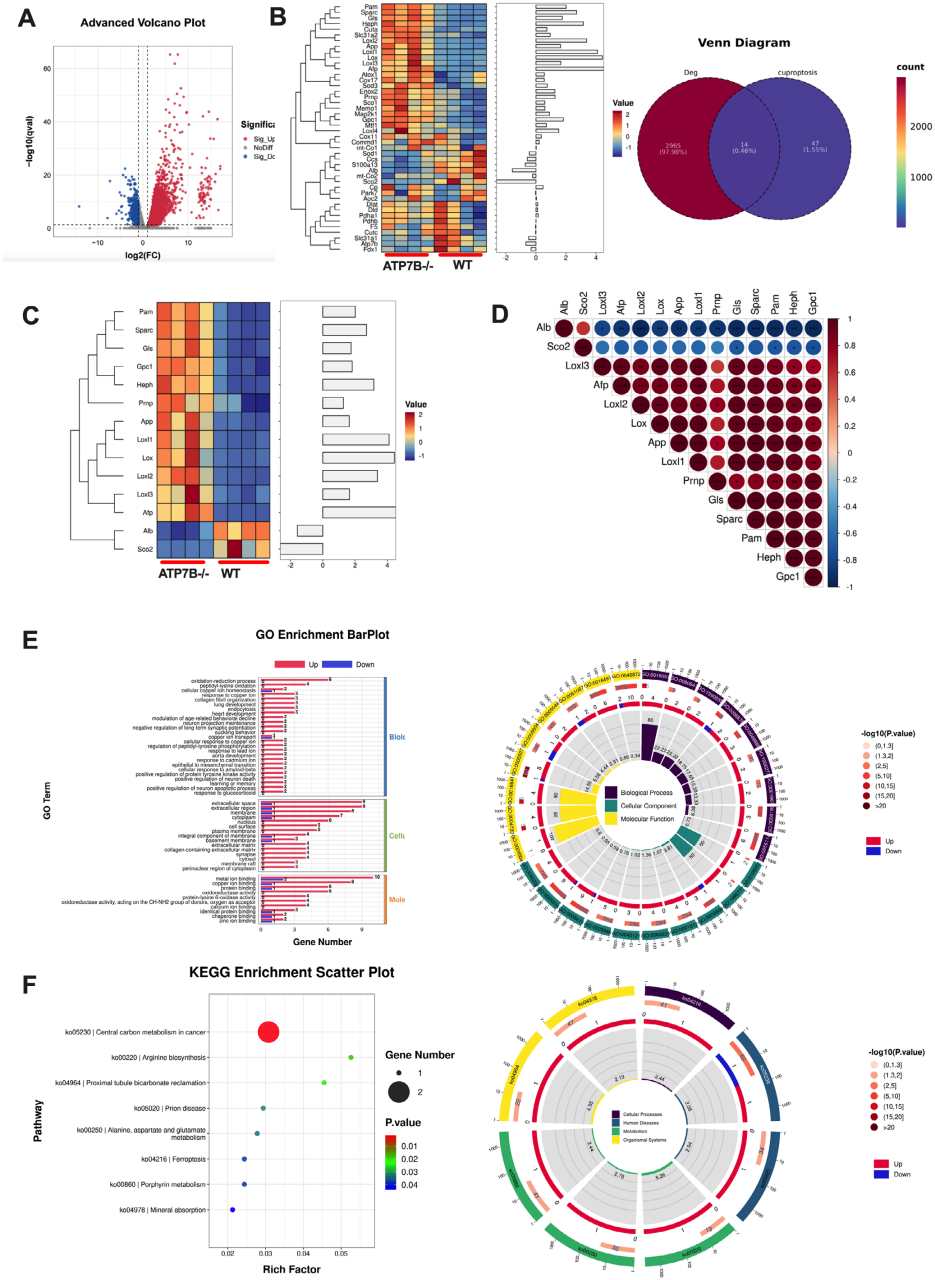
Transcriptomic analysis for screening potential cuproptosis‐related signalling molecules involved in the progression of Wilson disease. (A) Volcano plot of differentially expressed genes in WD and WT mice. (B) Heatmap showing the expression of all 61 cuproptosis‐related genes in ATP7B−/− and WT mice, Venn diagram illustrating the overlap between cuproptosis‐related genes and WD‐associated genes. (C) Heatmap of 14 differentially expressed cuproptosis‐related genes (DE‐CRGs). (D) Correlation analysis of DE‐CRGs in ATP7B−/− mouse samples. Red indicates a positive correlation, while blue represents a negative correlation. The colour intensity and size reflect the correlation strength. (E) Molecular function analysis of the 14 selected targets. (F) KEGG pathway enrichment analysis of the 14 selected targets. ATP7B−/−, ATP7B knockout mice; WT, wild‐type control mice.

To investigate cuproptosis‐associated genes in WD, we performed mRNA sequencing on liver tissues from four pairs of ATP7B−/− and C57BL/6 mice using the Illumina HiSeq 2000 (San Diego, CA) platform. Based on existing literature, 61 cuproptosis‐related genes (CRGs) were identified (Table S4). Differential gene expression analysis of the transcriptome data revealed 2979 differentially expressed genes (DEGs), with 2619 upregulated and 360 downregulated (Figure 5A). Venn diagram analysis was conducted to identify overlapping genes between WD‐associated gene sets and CRGs, revealing 14 differentially expressed CRGs (DE‐CRGs). Further analysis of their expression patterns and correlations between WD and WT groups showed that most genes were upregulated in the WD group, except for Alb and Sco2, which were downregulated(Figure 5B). Correlation analysis indicated strong associations among these genes, with the exception of Prnp (Figure 5C).

### 3.5 Enrichment Analysis of the Differential CRGs

We conducted GO and KEGG enrichment analyses on the 14 differentially expressed cuproptosis‐related genes (DE‐CRGs) to clarify their biological functions. The GO analysis encompassed three major categories: biological processes, cellular components and molecular functions. The findings revealed that these genes play roles in various biological processes, such as peptidyl‐lysine oxidation, cellular copper ion homeostasis, response to copper ions, collagen fibre organisation and redox regulation. The cellular component analysis highlighted their association with the extracellular space, extracellular region, basement membrane, extracellular matrix, collagen‐containing extracellular matrix and cell surface. In terms of molecular function, these genes were linked to copper ion binding, protein‐lysine 6‐oxidase activity, oxidoreductase activity (acting on CH‐NH_2_ group donors with oxygen as the acceptor), metal ion binding, molecular chaperone binding and fatty acid binding (Figure 5E).

Additionally, KEGG pathway analysis identified significant enrichment in multiple metabolic pathways, including central carbon metabolism in cancer, arginine biosynthesis, proximal tubule bicarbonate recovery, prion disease, alanine, aspartate and glutamate metabolism, porphyrin metabolism and ferroptosis (Figure 5F).

### 3.6 Screening of the Key Feature Genes

To identify core genes involved in Wilson disease (WD) and cuproptosis, we employed an integrated analytical strategy that combined protein–protein interaction (PPI) network analysis with two machine learning algorithms: least absolute shrinkage and selection operator (LASSO) regression and support vector machine–recursive feature elimination (SVM‐RFE).

**FIGURE 6:**
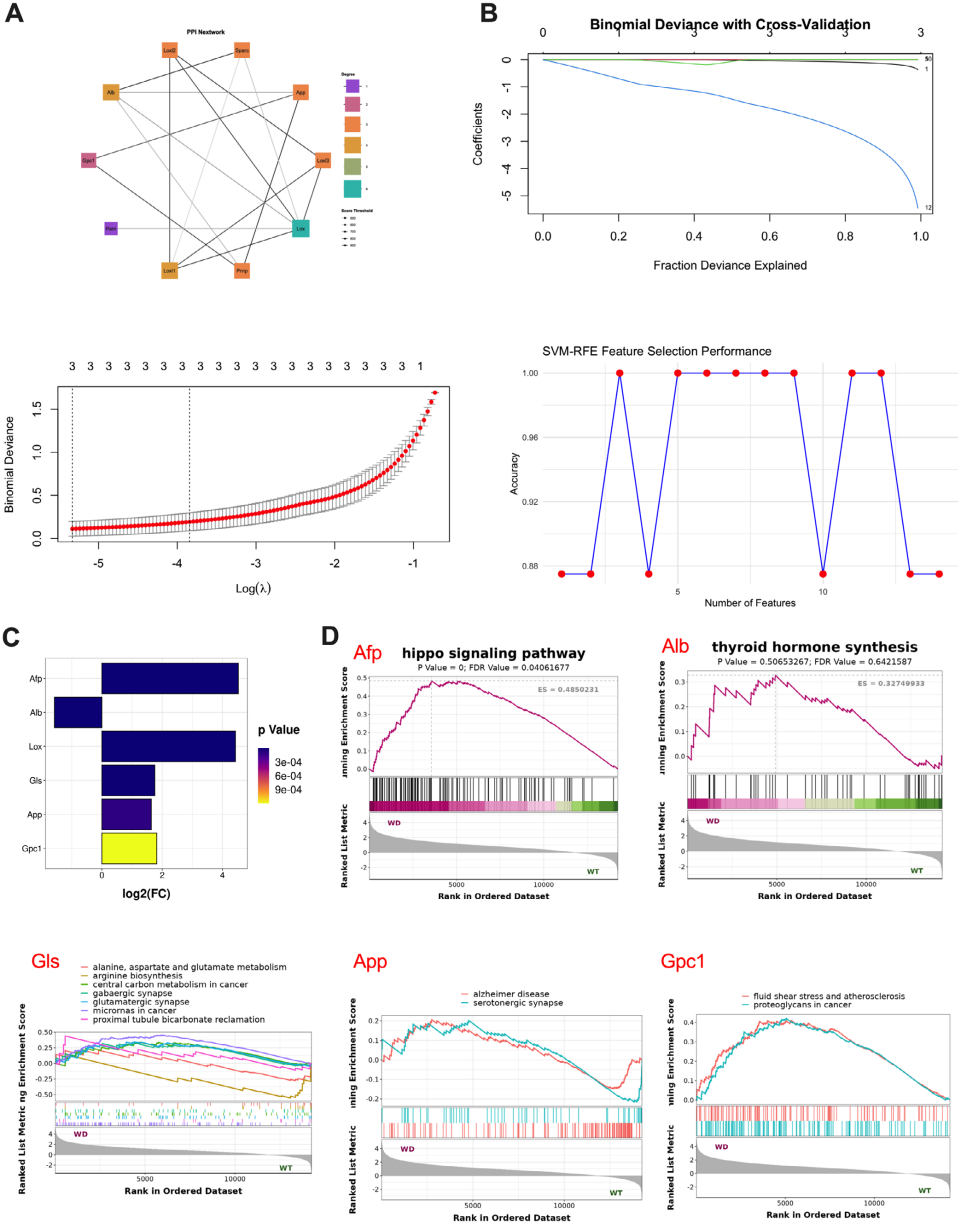
Identification of six DE‐CRGs as biomarkers for WD and single‐gene GSEA‐KEGG pathway analysis. (A) The top 10 hub genes identified in the PPI network. (B) LASSO algorithm for selecting 3 candidate genes and the SVM‐RFE algorithm for screening the 3 DE‐CRGs to identify the optimal combination of marker genes. (C) Bar chart of 6 differentially expressed genes (DEGs) showing log2 fold change (FC) values with *p*‐values indicated by colour gradient. (D) Single‐gene GSEA‐KEGG pathway analysis of Afp, Alb, Gpc1, Gls and App.

First, a PPI network was constructed based on the 14 differentially expressed cuproptosis‐related genes (DE‐CRGs) using the STRING database. This analysis highlighted Lox, App and Alb as central hub genes, suggesting their potential regulatory roles in the context of WD and copper‐induced cell death (Figure 6A).

Subsequently, LASSO regression was applied to identify the most informative predictors among the 14 DE‐CRGs, yielding Afp, Alb and Gpc1 as key candidates. Independently, SVM‐RFE analysis identified Afp, Gls and App as top‐ranking signature genes (Figure 6B).

By integrating the results from all three approaches, a total of six key genes including Lox, Afp, Alb, Gpc1, Gls and App, were consistently identified as being strongly associated with the pathogenesis and progression of WD (Figure 6C). These genes may serve as potential diagnostic biomarkers and mechanistic drivers of cuproptosis in WD and were subsequently subjected to further validation in both clinical samples and cellular models.

### 3.7 Gene Set Enrichment Analysis (GSEA) of Key Genes in ATP7B−/− Mice

The association of the total six key genes (Lox, Afp, Alb, Gpc1, Gls and App) with WD was further explored through single‐gene GSEA‐KEGG analysis. The results revealed the following associations: Lox was not enriched in the pathway. Afp was linked to the Hippo signalling pathway. Alb correlated with the Thyroid hormone synthesis pathway. Gls showed significant associations with multiple pathways, including alanine, aspartate and glutamate metabolism, arginine biosynthesis, central carbon metabolism in cancer, gabaergic synapse, glutamatergic synapse, microRNAs in cancer and proximal tubule bicarbonate reclamation. App was associated with the Alzheimer's disease and serotonergic synapse pathways. Gpc1 was linked to pathways related to fluid shear stress and atherosclerosis as well as proteoglycans in cancer. These findings highlight the complex roles these genes may play in the pathophysiology of WD (Figure 6D).

### 3.8 Validation of Key Genes in ATP7B−/− Mice and Functional Assessment via siRNAs Knockdown in ATP7B−/− HepG2 Cells

**FIGURE 7:**
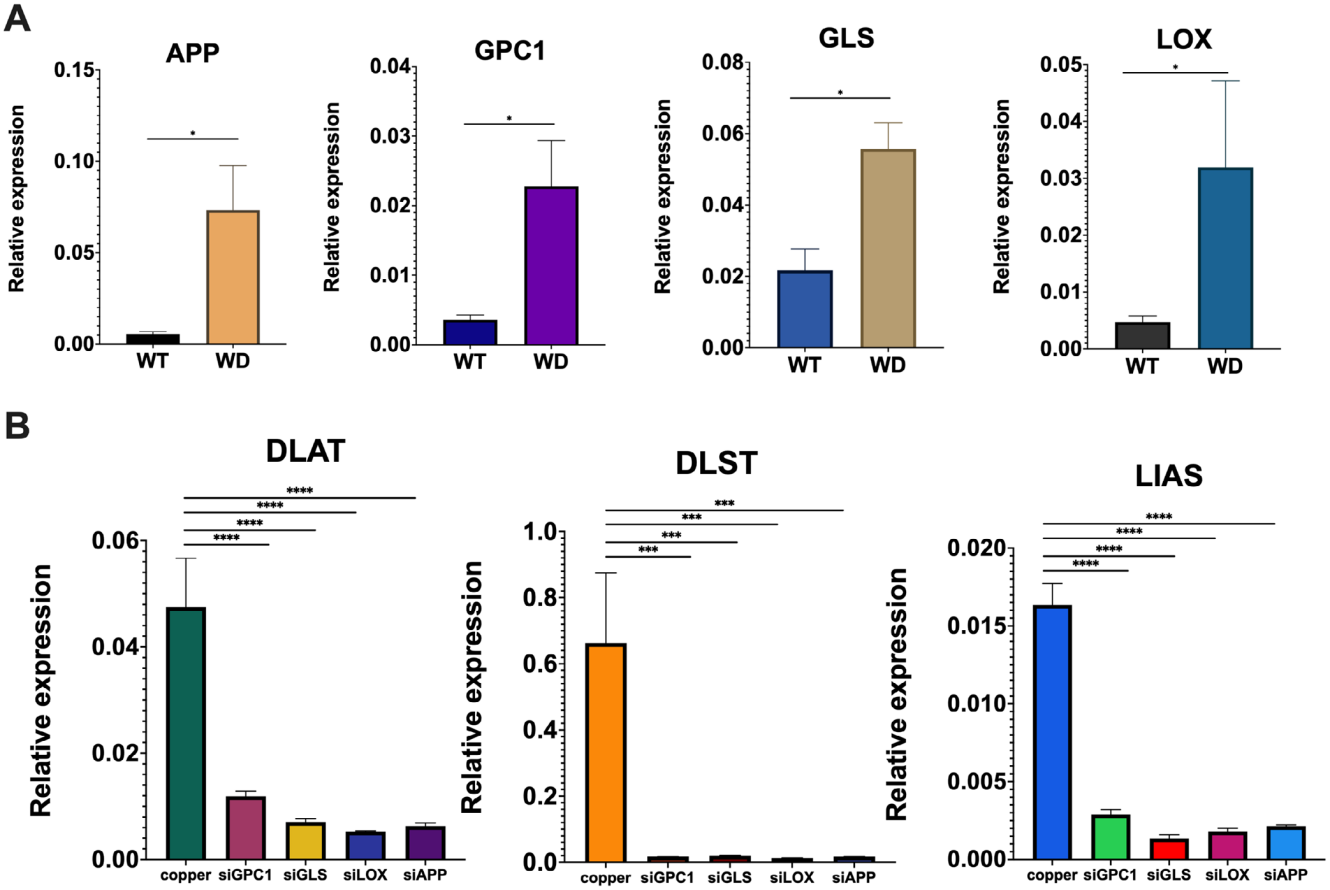
Expression analysis of hub genes and functional validation. (A) Relative hepatic expression levels of App, Gpc1, Gls and Lox in ATP7B−/− mice and WT mice, showing significant expression differences. (B) mRNA expression levels of DLAT, DLST and LIAS in ATP7B−/− HepG2 cells after transfection with siRNAs targeting GPC1, GLS, LOX and APP. Copper: ATP7B−/− HepG2 cells treated with 600 μmol/L CuSO_4_ alone; SiGPC1, SiGLS, SiLOX and SiAPP represent ATP7B−/− HepG2 cells subjected to siRNA‐mediated knockdown of GPC1, GLS, LOX and APP, respectively, followed by treatment with 600 μmol/L CuSO_4_. *p < 0.05, ***p < 0.001, ****p < 0.0001.

To further validate the relevance of the identified core genes in WD, we evaluated their expression in the ATP7B−/− mice model. Transcriptomic analysis revealed upregulation of Afp and downregulation of Alb, commonly measured serum indicators reflecting hepatocellular regeneration and injury. These changes mainly reflect liver stress rather than disease‐specific alterations and are therefore not discussed further. The focus of this study is on the identification and validation of novel therapeutic targets. To investigate the potential mechanistic roles of the other core genes, we analysed the hepatic expression of Lox, Gpc1, Gls and App in ATP7B−/− mice and wild‐type controls. Quantitative RT‐PCR analysis revealed that all four genes were significantly upregulated in ATP7B−/− mice (Figure 7A), indicating their possible involvement in the pathophysiological response to copper overload. What's more, transfection with siRNAs targeting GPC1, GLS, LOX and APP led to significant downregulation of cuproptosis‐related genes. Specifically, mRNA levels of DLAT, DLST and LIAS were markedly decreased in cells transfected with siGPC1, siGLS, siLOX or siAPP following treatment with 600 μM copper, compared with ATP7B−/− HepG2 cells without transfection. These results indicate that these hub genes play a functional role in regulating cuproptosis in hepatic cells. (Figure 7B).

## 4 Discussion

Wilson disease (WD) is a genetic disorder characterised by excessive copper accumulation, leading to hepatocellular damage [16]. Despite advances in understanding the pathophysiology of WD, the precise molecular mechanisms underlying copper‐induced hepatocellular damage remain incompletely understood. One emerging area of interest is cuproptosis, a novel form of copper‐induced cell death that has been implicated in mitochondrial dysfunction and cellular stress. By integrating in vivo and in vitro models with transcriptomic analysis, this study investigated the role of cuproptosis in WD‐related liver injury and identified key genes along with their associated signalling pathways. These findings offer new insights into potential therapeutic targets for WD.

Cuproptosis differs from known regulated cell death mechanisms and is characterised by the accumulation of fatty acylated proteins and the instability of iron–sulfur cluster proteins. As we know, WD is characterised by excessive copper accumulation in internal organs; some studies believe that the cellular effects of copper overload in WD resemble those induced by Cu^2+^ [17]. To date, only one study has measured key cuproptosis proteins in the serum of WD patients, revealing elevated levels of certain proteins [18]. However, no studies have directly demonstrated the role of cuproptosis in WD‐related hepatocyte damage. First, this study provides compelling evidence that copper overload induced cuproptosis contributes to liver damage in WD. In our in vivo ATP7B−/− mouse model, we observed significant liver damage, including hepatocyte swelling, necrosis and inflammatory infiltration, as well as marked elevations in serum markers of liver injury, such as AST, ALT and LDH. In vitro, we demonstrated a dose‐dependent relationship between CuSO_4_ treatment and cell damage, with increasing copper concentrations leading to reduced cell viability and heightened LDH release. These findings confirm that copper accumulation plays a crucial role in liver dysfunction in WD. Additionally, we showed that copper overload activates cuproptosis in both liver tissues of WD mice and ATP7B−/− HepG2 cells. This conclusion was supported by the upregulation of cuproptosis‐associated markers, such as FDX1, DLST, DLAT and LIAS. Furthermore, inhibiting cuproptosis mitigated cell damage, underscoring the critical role of cuproptosis in copper‐induced cell injury.

To elucidate the molecular mechanisms underlying cuproptosis in WD, we performed transcriptome sequencing and identified 14 differentially expressed cuproptosis‐related genes (DE‐CRGs) in WD. Using PPI protein interaction analysis, along with LASSO and SVM‐RFE algorithms, we identified six key cuproptosis‐related genes that emerged as central players in copper toxicity: Afp, Alb, Gpc1, Gls, Lox and App. In ATP7B−/− mice, the Afp, Gpc1, Gls, Lox and App genes were significantly upregulated, while Alb was markedly downregulated.

Among the cuproptosis‐related genes, Afp and Alb are commonly measured in clinical practice and reflect liver function, mainly indicating hepatocellular regeneration and liver injury, without specific significance for WD.

Notably, the other identified genes in ATP7B−/− mice were linked to various critical biological pathways. First, Gpc1‐encoded phosphatidylinositol proteoglycan is associated with fluid shear stress, atherosclerosis and proteoglycans in cancer, highlighting its significant role in vascular and tumour biology. Previous studies have shown that Gpc1 is highly expressed in HCC and is significantly associated with poor prognosis in HCC patients. Suppressing Gpc1 expression has been found to inhibit the proliferation, invasion and migration of HCC cells. Additionally, Gpc1 influences the malignant biological behaviour of HCC by regulating the Hippo signalling pathway [19]. Notably, the GSEA analysis in our study also identified an association between Afp expression and the Hippo signalling pathway. This finding is particularly meaningful given that the Hippo pathway has been extensively implicated in liver size regulation, regeneration, development, metabolism and homeostasis. Genetic studies in mouse and rat models have confirmed that dysregulation of this pathway is linked to the pathogenesis of common liver disorders, including fatty liver disease and liver cancer [20, 21]. Our results, which show concurrent upregulation of Gpc1 and Afp in WD liver tissue and their enrichment in Hippo‐related pathways, suggest that aberrant activation of this signalling axis may contribute to the hepatic injury and regenerative imbalance characteristic of WD. This convergence on the Hippo pathway offers novel insights into the molecular mechanisms underlying copper‐induced liver damage and raises the possibility of targeting this pathway for therapeutic intervention or biomarker development in WD. What's more, Gls encodes glutaminase, a key mitochondrial enzyme that catalyses the hydrolysis of glutamine to glutamate, playing a critical role in amino acid metabolism, nitrogen balance and cellular energy homeostasis. GSEA analysis in our study revealed that Gls is enriched in multiple metabolic and signalling pathways, particularly those related to amino acid metabolism, suggesting its involvement in broader metabolic reprogramming during disease progression. Recent studies have highlighted the role of Gls in the context of metabolic dysfunction‐associated fatty liver disease (MAFLD), where it contributes to hepatic inflammation and fibrosis by promoting glutaminolysis‐mediated oxidative stress, T cell activation and cuproptosis—a newly described form of regulated cell death driven by copper accumulation [22]. These mechanisms are thought to accelerate the transition from benign steatosis to hepatocellular carcinoma (HCC). Notably, similar pathological features, such as copper‐induced oxidative stress, chronic inflammation and progressive fibrosis, are hallmarks of WD. Our findings suggest that Gls may also play a significant role in the pathogenesis of WD, potentially through similar mechanisms involving copper‐driven metabolic stress and immune activation. Given the overlap in metabolic disturbance and cell death pathways between MAFLD and WD, it is plausible that Gls acts as a metabolic regulator that links copper overload with inflammatory and fibrotic responses in the WD liver. Moreover, the upregulation of Gls in WD models may represent a compensatory or maladaptive response to mitochondrial dysfunction and altered glutamine metabolism. These insights position Gls as a potential biomarker and therapeutic target in WD, warranting further investigation into its mechanistic role and the therapeutic implications of targeting glutaminase activity in copper‐associated liver diseases. App, the gene encoding amyloid precursor protein, is widely recognised for its association with neurodegenerative conditions, particularly Alzheimer's disease, where its cleavage product amyloid beta (Aβ) is a key pathological hallmark. In addition to its role in the central nervous system, App also participates in peripheral biological processes, including those in the liver, through its involvement in pathways such as serotonergic synapse signalling, oxidative stress regulation and intercellular communication. Emerging evidence has suggested that APP and its derivatives may exert hepatoprotective effects. Specifically, studies have demonstrated that APP can inhibit hepatic stellate cell activation and reduce extracellular matrix deposition, thereby mitigating liver fibrosis. Additionally, Aβ peptides have been reported to help maintain cell–cell adhesion and preserve the structural integrity of healthy hepatic tissue [23]. In our study, we observed a significant upregulation of App in the livers of ATP7B−/− mice, which may reflect an endogenous protective response to copper‐induced hepatotoxicity. Given the well‐established role of copper accumulation in promoting oxidative stress, mitochondrial damage and hepatocyte apoptosis in WD, the increased expression of App may represent a compensatory mechanism aimed at counteracting these pathological insults. The upregulation could also serve to modulate inflammatory responses or stabilise hepatic architecture in the face of chronic injury. This finding expands the relevance of App beyond neurodegeneration and positions it as a potentially important modulator of liver injury. Whether App upregulation in WD has functional consequences in attenuating fibrosis or preserving hepatocyte viability remains to be clarified. Further research is needed to determine whether targeting the App‐Aβ axis might offer therapeutic benefit or biomarker utility in the context of WD. Lysyl oxidase (Lox) family members are extracellular, copper‐dependent enzymes crucial for the cross‐linking of structural extracellular matrix (ECM) components. They are closely associated with the development of liver fibrosis and cancer. The expression of most Lox family members is upregulated in liver fibrosis of various etiologies, and inhibiting the entire Lox family can reduce fibrosis progression [24]. In our study, we observed a significant upregulation of Lox in the livers of ATP7B−/− mice, which suggests that copper accumulation in WD may drive hepatic fibrosis at least in part through the induction of LOX family members. This aligns with the established role of Lox in ECM remodelling and fibrotic progression.

These new insights significantly expand our understanding of the molecular underpinnings of copper toxicity in WD, offering a more nuanced and comprehensive view of disease pathogenesis. First and foremost, our findings underscore the critical role of cuproptosis, a newly defined copper‐dependent form of regulated cell death, in the development of WD. The dysregulation of cuproptosis‐related genes suggests that excess copper not only accumulates in hepatocytes but also actively induces cell death pathways that contribute to liver injury and progressive dysfunction. Beyond this, our analysis reveals that key cuproptosis‐related genes (Gpc1, Gls, App, Lox) are intricately involved in diverse biological processes, including the Hippo signalling pathway, amino acid metabolism, immune activation, neuronal signalling and extracellular matrix remodelling. These findings suggest that copper toxicity in WD disrupts not only mitochondrial function and redox homeostasis, but also initiates a broad spectrum of metabolic, inflammatory and fibrotic responses. Moreover, siRNA knockdown experiments confirmed the critical roles of Gpc1, Gls, Lox and App, highlighting their importance as potential therapeutic targets. Collectively, our study highlights cuproptosis as a central mechanism in WD and sheds light on novel molecular players and regulatory axes, paving the way for future mechanistic studies and the development of targeted therapeutic strategies.

Despite its strengths, this study has several limitations. First, while we identified several key genes associated with cuproptosis in the context of WD, the precise cellular and molecular mechanisms by which these genes contribute to liver injury, inflammation and fibrosis remain incompletely understood. Second, the current findings are largely based on transcriptomic analyses and correlation‐based interpretations; functional validation through in vivo experiments is essential to determine the causal roles of these genes and to elucidate their interactions within cuproptosis and related signalling pathways.

In conclusion, this study provides valuable new insights into the role of cuproptosis in the pathophysiology of WD, highlighting its pivotal contribution to liver injury. By identifying key cuproptosis‐related genes, we propose potential therapeutic targets for WD. Further research is essential to validate these findings in in vivo models to explore the therapeutic potential of modulating cuproptosis. Ultimately, this work may lay the foundation for more effective strategies to mitigate the harmful effects of copper overload in WD patients.

## Author Contributions

**Shan Tang:** data curation (lead), formal analysis (lead), methodology (equal), writing – original draft (lead). **Feng Ren:** conceptualization (equal), investigation (equal), project administration (equal), supervision (equal), writing – review and editing (equal). **Wei Hou:** data curation (equal), formal analysis (equal), investigation (equal), validation (equal). **Zihao Fan:** data curation (equal), formal analysis (equal), methodology (equal). **Yinkang Mo:** methodology (equal), visualization (equal). **Xianru Zhu:** investigation (equal), methodology (equal), visualization (equal). **Yaling Cao:** data curation (equal), methodology (equal), software (equal). **Ling Xu:** formal analysis (equal), methodology (equal), software (equal). **Sujun Zheng:** conceptualization (lead), funding acquisition (lead), project administration (lead), supervision (lead), writing – review and editing (lead).

## Ethics Statement

The animal experiments in this study were approved by the Animal Ethics Committee of Capital Medical University. All procedures in this study comply with the ethical standards of the Ethics Committee of the Institute of Beijing You'an Hospital of Capital Medical University and the Declaration of Helsinki (revised in 2013).

## Consent

The authors have nothing to report.

## Conflicts of Interest

The authors declare no conflicts of interest.

## Supporting information


**TABLE S1:** Guide RNA used in this work.
**FIGURE S1:** Overview of the dot plot and GC content distribution.
**TABLE S2:** The primers used in this work.
**TABLE S3:** The SiRNA used in this work.
**TABLE S4:** Cuproptosis‐related genes.

## Data Availability

The datasets used and/or analysed during the current study are available from the corresponding author on reasonable request.
