# Supplementary material for: Uncovering the Critical Role of Cuproptosis in Wilson Disease: Insights Into Potential Therapeutic Targets
Source: J Cell Mol Med. 2025 Nov 13;29(21):e70946. doi: 10.1111/jcmm.70946 (PMC12613164; doi:10.1111/jcmm.70946)
Supplement: Supplementary file 1 — TABLE S1: Guide RNA used in this work. FIGURE S1: Overview of the dot plot and GC content distribution. TABLE S2: The primers used in this work. TABLE S3: The SiRNA used in this work. TABLE S4: Cuproptosis‐related genes. [file JCMM-29-e70946-s001.doc]

**Supplementary Material**

**Elaboration of CRISPR Validation Details**

**Guide RNA (gRNA) Sequences and Design Rationale**

For the ATP7B gene knockout in HepG2 cells, we targeted Exon 2 of the canonical transcript ATP7B-201 (ENST00000242839.10), a region selected based on the following criteria: (1) proximity to the ATG start codon (Exon 1) to ensure frameshift mutations that disrupt protein translation; (2) small exon size and high gRNA scores (on-target efficiency) to maximize knockout efficiency; (3) avoidance of overlapping with other non-targeted transcripts (ATP7B-202, located downstream of the target region, thus not affected).

Three pairs of gRNAs were designed and validated, with sequences, on-target scores, and knockout parameters detailed below (Supplementary Table 1). On-target scores were evaluated using the Crispor algorithm (https://crispor.tefor.net/), where the first value represents the overall efficiency score (0-100, higher=more efficient) and the second value represents the specificity score (0-1, higher=less off-target risk). In addition, we performed dot plot and GC content distribution analyses to verify the suitability of the selected target region (exon 2 of ATP7B-201) and gRNA design. In the dot plot analysis, alignment of the target region (±800 bp flanking sequence) with itself showed only the expected diagonal line without off-diagonal signals, indicating the absence of complex or repetitive elements that could interfere with gRNA binding or Cas9 cleavage. Sliding window analysis demonstrated a stable GC content (average 47.39%), within the optimal range (40-55%) for PCR amplification and Sanger sequencing, ensuring reliable downstream clone screening and sequencing validation (Supplementary Figure 1).

**Supplementary Table 1. Guide RNA used in this work**

| **gRNA Pair** | **gRNA ID** | **Sequence (5′→3′)** | **On-target scores**  **(Efficiency; Specificity)** | **Knockout Size** | **CDS Knockout Size** |
| --- | --- | --- | --- | --- | --- |
| Pair 1 | gRNA1 | ATATCGGTGTCTTTGGCCGA AGG | 96; 0.67 | 130 bp | 130 bp |
|  | gRNA2 | TGGCTCCCCACCGAGAAACC AGG | 85; 0.72 | 130 bp | 130 bp |
| Pair 2 | gRNA3 | GCTCAATATCAATTGGTCCC AGG | 91; 0.68 | 71 bp | 71 bp |
|  | gRNA4 | TCATTCAGCCCGAAGACCTC AGG | 86; 0.76 | 71 bp | 71 bp |
| Pair 3 | gRNA5 | GCTATCGAGGCACTTCCACC TGG | 90; 0.71 | 109 bp | 109 bp |


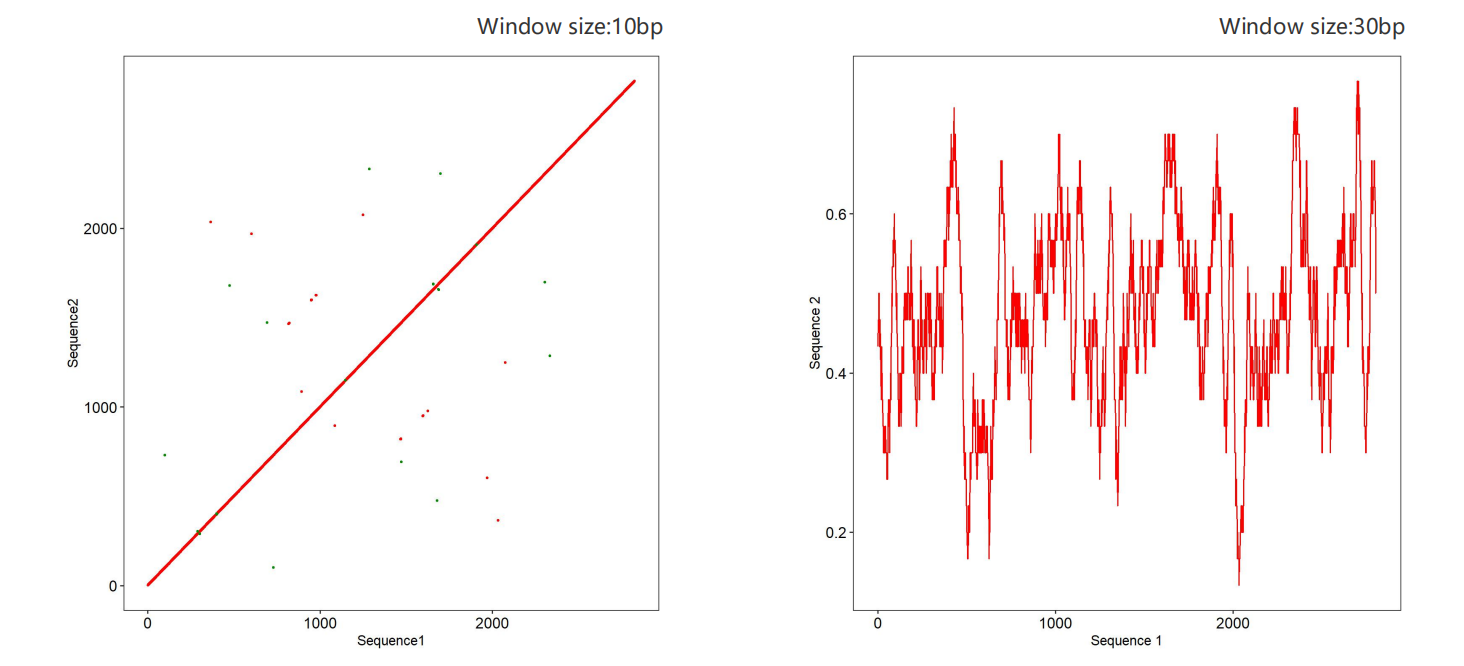


**Supplementary Figure 1. Overview of the Dot Plot and GC Content Distribution**

**Supplementary Table 2. The primers used in this work**

| **Primer** | **Forward** | **Reverse** |
| --- | --- | --- |
| DLST (mice) | CCGCTCGCTTTTCGCCTTCC | CTCCCACCTGACATCTCCCTCTG |
| DLAT (mice) | AGGAGACTTGCTGGAGAGATAGAG | GACTAACGAACACCTTCCCTTTG |
| LIAS (mice) | ATGCCAATGAGCCCGACAATACC | ATCCAGACAGACCACCTTCCC |
| FDX1 (mice) | ACTGCCATCTCCGTGGAC | CAACGTTCCCTCACACGCAC |
| GAPDH (mice) | ACCACAGTCCATGCCATCAC | TCCACCACCCTGTTGCTGTA |
| LIAS (human) | ACATGCCAAGAAGGTTCACGCC | CCAAAGGGCCACTTGCAGTAT |
| DLST (human) | TCGCTCTCCGCCTTCCAGAAG | CTCCCACCTGACATCTCCCTCTG |
| DLAT (human) | GCCGCCGCTATTACGTTCCC | AGGAGCCTGAGCAGAAGGTGTAG |
| ATP7B (human) | CTGGTGGTTGCTGGCTGAG | CTGGCATGGCTTCTCGTGATG |
| GAPDH (human) | TCTAGAGCTAGCGAATTCGCCACCATGCCTGAGCAGGAGAGACAG | CCTTCTCAGCCACAGCAACCAC |

**Supplementary Table 3. The SiRNA used in this work**

| **Primer** | **SiRNA sequence** |
| --- | --- |
| GPC1-1123-s | UUC ggA gAC CAg CUU CUC C/dT//dT/ |
| GPC1-389-s | UCU gCg UgU ACA gCU CUC C/dT//dT/ |
| GPC1-315-s | Uag AAa Uag UCA UCa AAa C/dT//dT/ |
| GLS-695-s | gAg UUA UAU gAA AgU gCU A/dT//dT/ |
| GLS-573-s | gUg UCA UgC UAg ACA AAg A/dT//dT/ |
| GLS-545-s | AgA UUA ACU CUU CAA ACA A/dT//dT/ |
| LOX-550-s | CAA CUA CUA CgA UAC UUA U/dT//dT/ |
| LOX-553-s | CUA CUA CgA UAC UUA UgA A/dT//dT/ |
| LOX-862-s | gCA CAg UUg UCA UCA ACA U/dT//dT/ |
| APP-1037-s | CAA AgU UUA CUC AAg ACU A/dT//dT/ |
| APP-117-s | gAC UgA ACA UgC ACA UgA A/dT//dT/ |
| APP-421-s | ggA UgU UUg CgA AAC UCA U/dT//dT/ |

**Supplementary Table 4. Cuproptosis-related genes**

| Atp7a | Slc31a1 | Map2k1 | Cox11 | Slc31a2 | Cox17 | Aoc2 |
| --- | --- | --- | --- | --- | --- | --- |
| Sco1 | Aoc3 | Sco2 | S100a5 | S100b | Atox1 | Ccs |
| S100a13 | Heph | mt-Co1 | Atp7b | Hephl1 | mt-Co2 | Aoc1 |
| Park7 | Cuta | Cp | App | Moxd1 | Dbh | Gpc1 |
| Enox1 | Prnp | Commd1 | Enox2 | S100a12 | Cutc | Lox |
| Snca | Memo1 | Loxl1 | Tyr | Sod1 | Loxl2 | Tyrp1 |
| Ltf | Loxl3 | Loxl4 | Pam | Sod3 | Afp | Alb |
| F5 | Mt3 | Mt4 | Sparc | Cdkn2a | Fdx1 | Dld |
| Dlat | Gls | Mtf1 | Pdha1 | Pdhb |  |  |
